# The role of AIM2 in inflammation and tumors

**DOI:** 10.3389/fimmu.2024.1466440

**Published:** 2024-11-12

**Authors:** Dalang Yu, Siping Zheng, Lina Sui, Yuling Xi, Tiansheng He, Zhiping Liu

**Affiliations:** ^1^ School of Basic Medicine, Fuzhou Medical College of Nanchang University, Fuzhou, Jiangxi, China; ^2^ School of Graduate, Gannan Medical University, Ganzhou, Jiangxi, China; ^3^ School of Basic Medicine, Gannan Medical University, Ganzhou, Jiangxi, China

**Keywords:** AIM2, inflammasome, inflammation, tumor, cytokine

## Abstract

Absent in melanoma 2 (AIM2) serves as an intracellular nucleic acid sensor that predominantly detects double-stranded DNA (dsDNA) within the cells. This detection initiates the assembly of inflammasome and activates the inflammasome signaling cascade, resulting in the production of inflammatory mediators and the cleavage of Gasdermins. Consequently, these processes culminate in inflammatory responses and pyroptotic cell death. AIM2 plays a pivotal role in modulating inflammation and tumorigenesis, functioning through both inflammasome-dependent and independent mechanisms. Its influence on the host immune response is dual-faceted, exhibiting both promotive and inhibitory effects in the contexts of inflammation and tumors. These effects are predominantly contingent upon the specific cell type expressing AIM2 and the nature of the host’s disease. This article seeks to review the latest advancements in understanding the cell-specific functions of AIM2 in inflammation and tumorigenesis, with the objective of offering insights for further research on AIM2 and informing the development of targeted therapeutic strategies for clinical application.

## 1 Introduction

Absent in melanoma 2 (AIM2), a member of the PYHIN protein family, is predominantly localized in the cytoplasm. Structurally, it comprises an N-terminal pyrin domain (PYD) and a C-terminal hematopoietic interferon-induced nuclear (HIN) domain (1). Through its HIN domain, AIM2 primarily recognizes microbial DNA, endogenous DNA from animal cells, and synthetically generated double-stranded DNA (dsDNA), which facilitates the assembly of AIM2 oligomers. Simultaneously, the PYD domain plays a crucial role in the recruitment and binding of the apoptosis-associated speck-like protein containing a CARD (ASC), which itself is composed of an N-terminal PYD domain and a C-terminal CARD domain. Through the integration of its own CARD domain with pro-Caspase-1, AIM2 facilitates the assembly of inflammasomes. In the absence of dsDNA within the cytoplasm, AIM2 molecules engage in self-association via AIM2-AIM2 interactions, thereby maintaining a basal low concentration state (2). Upon the intrusion of dsDNA into the cytosol, there is an upregulation of AIM2 gene expression, resulting in an elevated local concentration. This upregulation enables AIM2 to recognize dsDNA and rapidly form the inflammasome, subsequently activating downstream signaling molecules within the pathway and triggering an inflammatory response (3, 4).

Consequently, a comprehensive examination of AIM2’s involvement in inflammation and tumorigenesis is instrumental in devising novel strategies to modulate AIM2 activity and, by extension, the host immune response. This understanding is crucial for advancing the clinical development of precision-targeted therapies aimed at treating related pathologies. Nonetheless, there remains a deficiency in systematic evaluations of the cell-specific mechanistic roles of AIM2 in these contexts. This review article addresses recent progress in elucidating the cell-specific functions of AIM2 within inflammatory and tumor environments.

## 2 AIM2 related signaling pathways

### 2.1 Classic signaling pathway of the AIM2 inflammasome activation

The PYD and HIN domains of AIM2 typically possess the ability to self-oligomerize; however, in conjunction with ASC, they fail to form the requisite oligomers necessary for initiating downstream signaling. Upon exposure to DNA viruses or transfection with DNA analogues, AIM2 recognizes and binds to dsDNA in the cytoplasm. This binding occurs through the positively charged HIN domain, which engages with the sugar-phosphate backbone of the dsDNA via electrostatic interactions (2, 4). When the length of dsDNA exceeds the threshold of 70bp, the affinity increases sharply, exceeding the AIM2-AIM2 interaction affinity, until it reaches its maximum value at approximately 280bp (2). When AIM2 is activated, it releases from its oligomeric state and recruits ASC through PYD-PYD interactions. Subsequently, ASC interacts with Pro-Caspase-1, another protein possessing a CARD domain, via CARD-CARD domain interactions, which assembles the inflammasome complex comprising AIM2, ASC, and Pro-Caspase-1 (AIM2-ASC-Pro-Caspase-1). Subsequently, Caspase-1 catalyzes the processing of Pro-IL-18, Pro-IL-1β, and Gasdermins, leading to the maturation and secretion of the pro-inflammatory cytokines IL-18 and IL-1β, in addition to inducing cell pyroptosis (4, 5).

**Figure 1:**
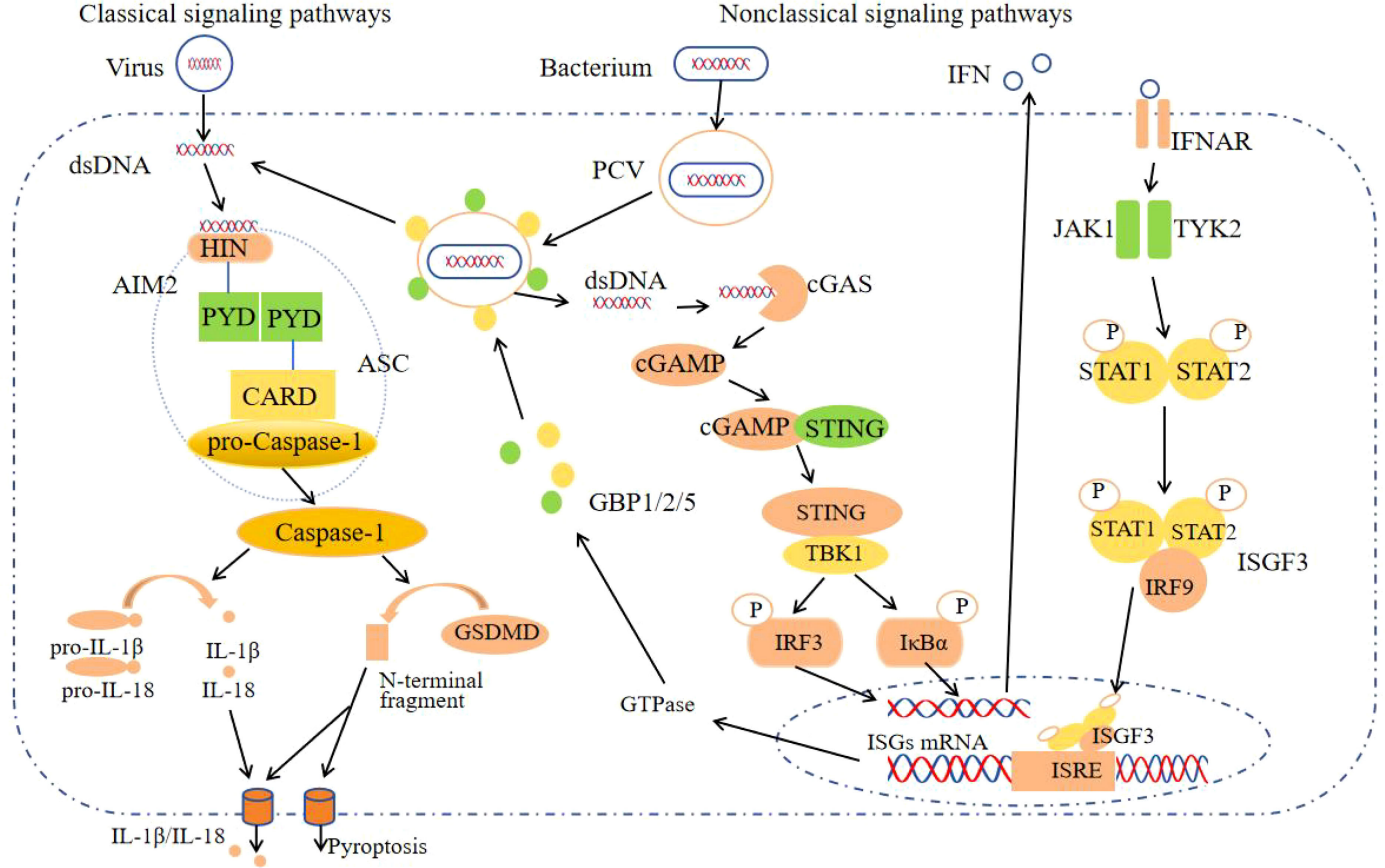
The AIM2 inflammasome activation signaling pathway. After AIM2 recognizes the virus dsDNA in the cytoplasm, it forms AIM2-ASC-Pro-Caspase-1, activates Caspase-1, cleaves Pro-IL-18, Pro-IL-1β and GSDMD, secretes IL-18, IL-1β and causes pyroptosis, which is the classic signaling pathway; Non-classical signaling pathways: GBPs cleave PCV, expose bacterial dsDNA, activate the classical signaling pathway of the AIM2 inflammasome after recognition by AIM2, and the remaining dsDNA generates GBPs through the cGAS-STING signaling pathways for bacterial lysis.

Gasdermins constitute a family of pore-forming protein effectors that can initiate cell death. They are characterized by an N-terminal cytotoxic domain and a C-terminal inhibitory domain. In mammals, gasdermin D (GSDMD) is a notable member of this family and contains a cleavage site for Caspase-1 (6). Upon cleavage by activated Caspase-1, the N-terminal domain of GSDMD inserts into the cell membrane, forming large oligomeric pores. This pore formation disrupts cellular ion homeostasis and induces pyroptosis (5) (**Figure 1**). This process occurs rapidly and operates independently of type I interferon (IFN) activity (7). Numerous DNA viruses, including mouse cytomegalovirus, vaccinia virus, and human papillomavirus, activate the AIM2 inflammasome through this mechanism (8).

### 2.2 Non-classical signaling pathway of the AIM2 inflammasome activation

During bacterial infection, the intracellular receptor AIM2 detects the nucleic acids of bacteria, necessitating initial bacterial lysis. This lytic process is facilitated by interferon-induced guanylate-binding proteins (GBPs), such as GBP1, GBP2, and GBP5 in humans, among other variants (9, 10). Upon invasion of host cells, pathogenic bacteria form pathogen-containing vacuoles (PCVs). Subsequently, GBPs disrupt the PCVs, releasing bacterial dsDNA into the cytoplasm, where it is recognized by AIM2. This recognition activates the canonical signaling pathway of the AIM2 inflammasome. Furthermore, bacterial DNA present in the cytoplasm also stimulates the production of IFN-I through various intracellular receptors, including cyclic GMP-AMP synthase (cGAS) (11). Upon activation by intracellular dsDNA, cGAS catalyzes the synthesis of cyclic GMP-AMP (cGAMP). Subsequently, cGAMP binds to the stimulator of interferon genes (STING) protein located in the endoplasmic reticulum, inducing conformational changes, oligomerization, and translocation of STING. This process facilitates the aggregation of STING with TANK-binding kinase 1 (TBK1), forming the STING-TBK1 complex. TBK1 is capable of phosphorylating IRF3 to induce the production of IFN-I. Additionally, it can phosphorylate inhibitors of IκBα, facilitating the release of NF-κB into the nucleus, which subsequently regulates the transcription of inflammatory mediators and the expression of auxiliary IFN-I (11). Ultimately, IFNs activate Janus kinase 1 (JAK1) and tyrosine kinase 2 (TYK2) through their interaction with the IFNAR, leading to the phosphorylation of STAT1 and STAT2. The phosphorylated STAT1 and STAT2 then associate with IFN regulatory factor 9 (IRF9) to form the trimeric IFN-stimulated gene factor 3 (ISGF3) complex. The activated transcription factor ISGF3 translocates to the nucleus, where it induces the transcription of interferon-stimulated genes (ISGs) by binding to interferon-stimulated response elements (ISRE). Among these ISGs are a series of GTPase enzymes, including GBPs (12, 13). These enzymes facilitate the extensive release of bacterial DNA into the cytoplasm, allowing for its recognition by AIM2 (**Figure 1**).

### 2.3 Other related signaling pathways of AIM2

**Figure 2:**
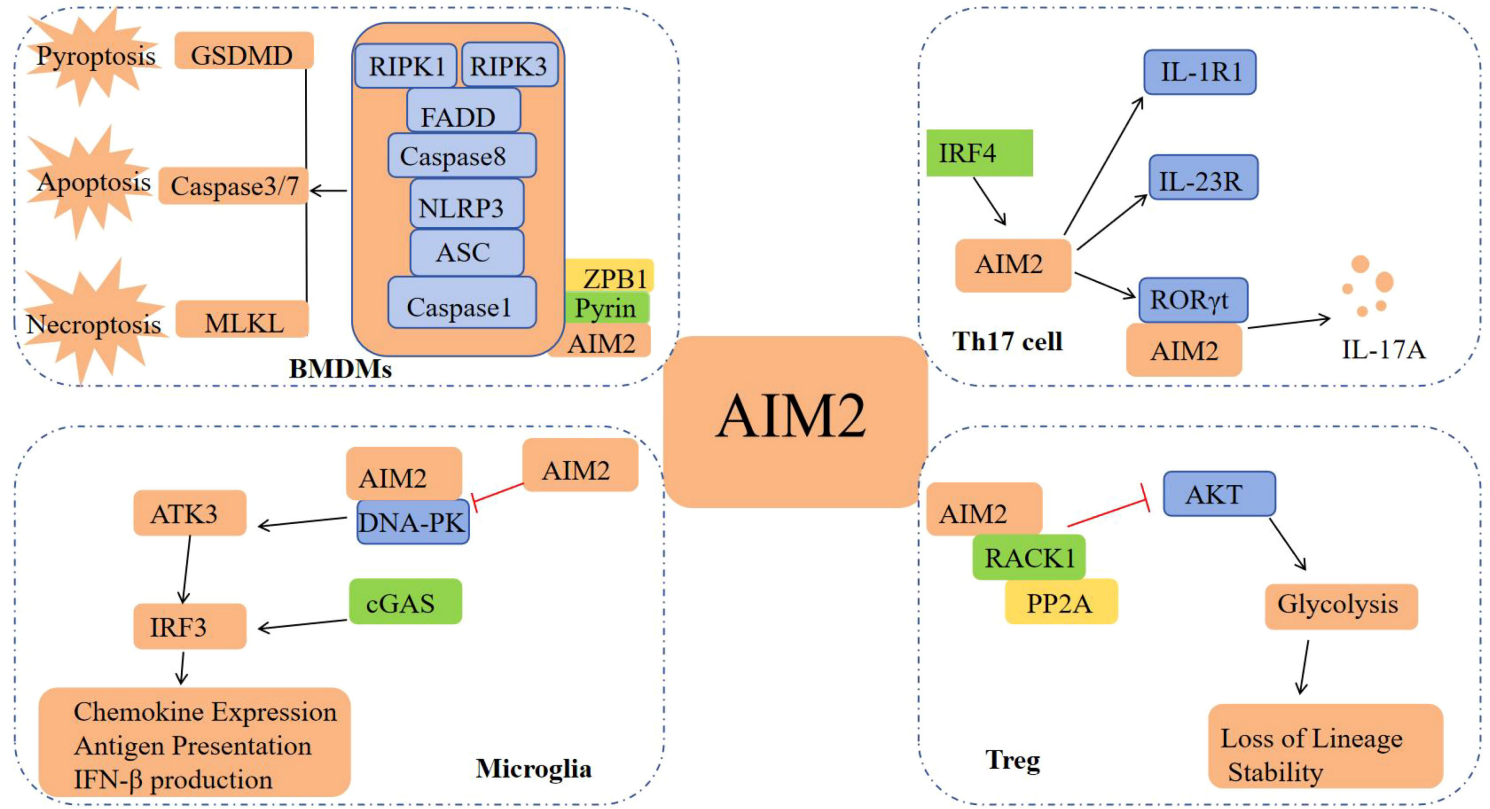
Other signaling pathways of AIM2. In BMDMs, AIM2 forms PANoptosome with Pyrin, ZBP1, ASC, etc., inducing cell pyroptosis, apoptosis, and necrosis; In Th17 cells, the interaction between AIM2 and RORγt promotes Th17 differentiation and IL-17A production; In Microglia, AIM2 inhibits DNA-PK and reduces the production of chemokines and IFN-I; In Treg cells, AIM2 inhibits AKT activation and maintains the lineage stability of Treg cells.

In addition to its extensively characterized involvement in the inflammasome signaling pathway, AIM2 also modulates the innate immune sensors Pyrin and ZBP1 by forming multiprotein complexes, known as PANoptosomes, which include ASC, Caspase-1, NLRP3, Caspase-8, RIPK3, RIPK1, and FADD. These complexes concurrently engage in Caspase-1 and GSDMD-mediated pyroptosis, Caspase-3/7-mediated apoptosis, and MLKL-mediated necrosis, thereby driving inflammatory signaling and PANoptosis (14). Furthermore, AIM2 possesses signaling pathways that operate independently of its function as a DNA receptor and its role in inflammasome activation. Recent studies have identified AIM2 as a regulator of the effector program in Th17 cells, achieved through the upregulation of RORγt, IL-1R1, and IL-23R expression. AIM2 interacts with RORγt to facilitate Th17 differentiation and IL-17A production. Concurrently, IRF4 modulates AIM2 expression during Th17 cell differentiation, thereby playing a crucial role in the pathogenesis of colitis (15). In a similar manner, inflammation-independent functions are mediated through two distinct signaling pathways. In microglia, AIM2 acts to inhibit DNA-dependent protein kinase (DNA-PK), thereby suppressing the activation of AKT3. This inhibition leads to a reduction in the phosphorylation of the transcription factor IRF3, which is a component of the cGAS signaling pathway. Consequently, this cascade results in a diminished production of chemokines and IFN-I, as well as a reduced expression of microglial antigen-presenting molecules (16); In Treg cells, AIM2 interacts with RACK1 and phosphatase PP2A to attenuate AKT activation, consequently diminishing mTOR and Myc activation. This shift promotes a metabolic transition towards oxidative phosphorylation, thereby preserving the lineage stability of Treg cells (16) (**Figure 2**). Ongoing intensive research on AIM2 is expected to uncover additional signaling pathways independent of the AIM2 inflammasome.

## 3 Role of AIM2 in inflammation

Considering that AIM2 does not differentiate between self and non-self dsDNA, it is widely posited that the activation of the AIM2 inflammasome underlies the pathogenesis of inflammation and related inflammatory diseases. To substantiate this hypothesis, extensive research has been undertaken to investigate the pathogenic role of AIM2 and its inflammasome in the context of inflammation and inflammatory diseases.

### 3.1 Role of AIM2 in autoimmune diseases

#### 3.1.1 The pro-inflammatory role of AIM2 in autoimmune diseases

In rheumatoid arthritis (RA), patients exhibited reduced levels of AIM2 in their sera compared to healthy individuals, whereas levels of ASC, Caspase-1, and IL-1β are elevated (17). This phenomenon might be attributed to the diminished expression of AIM2 in specific immune cells, such as CD14^+^ cells, in RA patients (18). This reduced expression could be associated with impaired DNA repair mechanisms (19) and an upregulation of molecules like POP3 (LST8 homolog), which specifically inhibits the AIM2 inflammasome (20). Conversely, the expression of AIM2, ASC, Caspase-1, and IL-1β was increased in the synovium of RA patients relative to those with osteoarthritis (17). Specifically, the expression of AIM2 was markedly elevated in fibroblastic synoviocytes (FLS), contributing to cartilage destruction, as reported in a previous study (21). Furthermore, elevated levels of IL-1β, secreted following the activation of the AIM2 inflammasome in monocytes, macrophages, and dendritic cells, were also implicated in the degradation of bone joints in RA (17).

In a similar manner, in gouty arthritis induced by monosodium urate (MSU) crystals, the interaction between MSU and macrophages resulted in the formation and activation of the inflammasome. Specifically, the AIM2 inflammasome regulated the secretion of inflammatory cytokines, such as IL-1β and IL-18, thereby mediating inflammation and pyroptosis, as well as exacerbating pain and inflammation in gouty arthropathy (22). These findings indicated that AIM2 might contribute to the disruption of articular cartilage and the proliferation of FLS by producing inflammatory cytokines in various cell types, thus playing a critical role in the onset and persistence of destructive arthritis. Previous studies had demonstrated that AIM2 contributed to pro-inflammatory pathogenic effects via the inflammasome signaling pathway in conditions such as primary Sjogren’s syndrome (pSS) (23), dry eye disease (DED) (24), and experimental autoimmune uveitis (EAU) (25). Additionally, some researchers had identified that AIM2 influenced the function of RA FLS by promoting AKT phosphorylation (26). However, the precise mechanism underlying this process remains unclear, and it is uncertain whether it is directly associated with the AIM2 inflammasome.

Moreover, AIM2 is capable of facilitating the advancement of inflammation and inflammatory diseases through non-classical or non-inflammasome signaling pathways. In individuals with psoriasis, neutrophil extracellular traps (NETs) not only activated AIM2 inflammation via traditional signaling pathways but also induced the AIM2 inflammasome through the p38/MAPK signaling cascade in a manner independent of dsDNA, resulting in the production of IL-1β (27). AIM2 was observed to enhance the expression of X-linked apoptosis inhibitor protein (XIAP), facilitate the secretion of IFN-γ in keratinocytes, and mediate the immune response of these cells (27). In the context of T follicular helper (TFH) cell-mediated systemic lupus erythematosus (SLE), AIM2 was found to promote TFH cell differentiation and IL-21 production via the c-myofascial fibrosarcoma (c-MAF) signaling pathway. Concurrently, IL-21 augmented AIM2 expression by increasing the enrichment of 10-11 translocation 2 (TET2) in the AIM2 promoter region, thereby further advancing the progression of SLE (28). A separate study demonstrated that AIM2 facilitated the differentiation of Th17 cells and the production of IL-17A, while also upregulating Th17-associated proteins such as RORγt, IL-1R1, and IL-23R, thereby contributing to the development of inflammation (15). Furthermore, various forms of cell death, including apoptosis, pyroptosis, and necrosis, had been identified in RA (29). Key molecules involved in pyroptosis and necrosis were upregulated in several cell populations associated with RA. For instance, the expression levels of RIPK1, RIPK3, and phosphorylated MLKL (p-MLKL) were elevated in both an *in vivo* arthritis experimental animal model and *in vitro* acid-induced chondrocytes (30, 31). The data indicated the potential existence of signaling pathways in RA that operate independently of the AIM2 inflammasomes in exerting pro-inflammatory effects. Nevertheless, research on AIM2-mediated PANoptosis in the context of RA remains limited, highlighting the need for further investigation in this promising area of study.

#### 3.1.2 The anti-inflammatory role of AIM2 in autoimmune diseases

Contrary to prior expectations, AIM2 demonstrated the capacity to mitigate inflammation and, in certain instances, impede the progression of inflammatory diseases. Recent studies had revealed that AIM2 enhanced the stability of Treg cells by attenuating AKT phosphorylation, mTOR and MYC signaling, and glycolysis, while promoting lipid oxidative phosphorylation, thereby ameliorating autoimmune diseases (32). Similarly, in the context of rhabdomyolysis induced acute kidney injury (RIAKI), it was observed that macrophages deficient in AIM2 did not regulate dsDNA-induced pyroptosis but instead activated alternative DNA sensing pathways, such as STING, resulting in heightened inflammation and fibrosis (33). These findings suggested that AIM2-induced pyroptosis played a crucial role in modulating inflammatory responses. The swift degradation of macrophages triggered by AIM2 might curtail the activity of viable cells, thereby reducing the subsequent production of inflammatory cytokines. Subsequent experiments within this study demonstrated that AIM2-induced pyroptosis effectively inhibited the pro-inflammatory STING/CXCL10/CXCR3 signaling pathway both *in vivo* and *in vitro*, consequently attenuating inflammation associated with acute kidney injury (33).

### 3.2 Role of AIM2 in the inflammation of cardiovascular diseases

In the initial phases of atherosclerosis, monocytes attracted by dysfunctional endothelial cells differentiated into macrophages, which subsequently internalized modified lipoproteins and transformed into foam cells within plaques (34). During this process, the AIM2 inflammasome in macrophages not only contributed to the induction of vascular cell adhesion protein-1 (VCAM-1) expression in atherosclerosis (35), but also limited cholesterol transport and facilitated foam cell formation (36, 37). In vascular smooth muscle cells (VSMCs), AIM2 had been shown to exacerbate the progression of atherosclerosis and accelerate plaque formation by facilitating endothelial cell migration and apoptosis (38). Furthermore, the AIM2 inflammasome might activate TGF-β, SMAD2, and SMAD3, leading to increased expression of matrix metalloproteinase-2 (MMP2) within atherosclerotic plaques and further promoting VSMC migration (39). However, the direct interaction between AIM2 and TGF-β remains unclear. In the advanced stages of atherosclerosis, the AIM2 inflammasome within macrophages contributed to the formation of necrotic cores in atherosclerotic lesions. While both the NLRP3 and AIM2 inflammasomes can be activated in macrophages, the AIM2 inflammasome played a more significant role in promoting atherogenesis (40). Importantly, the AIM2 inflammasome was consistently expressed in healthy vascular wall cells and its expression increased with the progression of atherosclerotic burden (41). AIM2 contributed to the acceleration of atherosclerosis and enhanced plaque instability, particularly in the context of clonal hematopoiesis associated with the JAK2VF mutation (40).

Similarly, in conditions such as coronary artery disease (CAD) (42) and diabetes (43), the AIM2 inflammasome activation exacerbated atherosclerosis. In the context of diabetic cardiomyopathy, AIM2 activation had been shown to induce a pro-inflammatory phenotype in macrophages, thereby enhancing the production of TNF-α and IL-1β, as well as activating and promoting the assembly of the NLRC4 inflammasome in cardiomyocytes (44). The assembly of the NLRC4 inflammasome subsequently initiated the IRF1/NF-κB signaling pathway, culminating in cardiac hypertrophy and remodeling (44). These findings collectively suggest that the advancement of inflammation in cardiovascular diseases may be contingent upon the pro-inflammatory role of AIM2 activation within the cells of the affected tissue.

### 3.3 Role of AIM2 in inflammation in stroke and neuro-degenerative diseases

Stroke, predominantly ischemic stroke (IS), is a life-threatening medical condition. Previous research had demonstrated that a deficiency in AIM2 contributed to the preservation of blood-brain barrier (BBB) integrity following ischemic or hypoxic events. This protective effect was achieved through the inhibition of STAT3 activation, the upregulation of tight junction (TJ) protein expression, and the downregulation of ICAM-1 expression (45). Nonetheless, this study was limited to examining the role of AIM2 in ischemic stroke using whole-gene knockout mice and did not clarify whether the AIM2 inflammasome was implicated. Following cerebral ischemia/reperfusion (I/R) in the rat brain, activation of the AIM2 inflammasome was observed, which was associated with elevated expression levels of AIM2, ASC, IL-1β, and IL-18 (46). The onset of IS and subsequent reoxygenation resulted in excessive oxidative stress within the mitochondria, leading to the accumulation of ROS and further mitochondrial damage. This damage facilitated the leakage of dsDNA into the cytoplasm, thereby activating the AIM2 inflammasome (47). The activation of the AIM2 inflammasome subsequently triggered the secretion of inflammatory cytokines in neurons and the opening of the Pannexin-1 pore, ultimately inducing neuronal cell death (48). Significantly, the AIM2 inflammasome facilitated the removal of defective neurons through the induction of localized cell death, thereby contributing to normal neurodevelopmental processes (49). Consequently, the AIM2 inflammasome serves a dual function in both physiological and pathological neuronal contexts, playing a critical role in maintaining neuronal homeostasis. Furthermore, the release of intracellular dsDNA following tissue damage initiated the assembly of the AIM2 inflammasome and the subsequent release of IL-1β in myeloid cells. This process enhanced the expression of Fas ligand in monocytes, which subsequently interacted with Fas receptors on T cells, resulting in T cell apoptosis (50). Therefore, the activation of AIM2 might exacerbate stroke pathology by inducing excessive T cell death. Correspondingly, research had demonstrated that the activation of the AIM2 inflammasome in astrocytes (46), microglia (51), macrophage (51), and neutrophil (52) contributed to a pro-inflammatory response, thereby worsening stroke conditions.

Notably, the AIM2 inflammasome activation in astrocytes and microglia has also been implicated in the exacerbation of neuroinflammation associated with Alzheimer’s disease (AD) (53). In animal models of experimental autoimmune encephalomyelitis (EAE) and Parkinson’s disease (PD), AIM2 molecules in microglia had been observed to negatively regulate the disease independently of inflammasome activation. Within microglia, AIM2 molecules inhibited DNA-PK-mediated AKT3 recruitment and phosphorylation, thereby limiting the transcription of interferon regulatory factor 3 (IRF3) and inflammatory cytokines, and providing neuroprotection (54, 55). These findings suggested that AIM2 exhibited both pro-inflammatory and anti-inflammatory roles in microglia, highlighting its complex functional dynamics. This phenomenon may be associated with the pathogenesis of inflammation across various diseases; however, the precise mechanisms by which AIM2 modulates pro-inflammatory or anti-inflammatory responses remain unclear and warrant further comprehensive investigation.

### 3.4 Role of AIM2 in the inflammation of other diseases

Research had demonstrated that elevated expression of AIM2 was correlated with an increased risk of inflammation in oral diseases (56). Specifically, overexpression of AIM2 significantly enhanced the activity of Caspase-1 and the secretion of IL-1β in human dental pulp cells (HDPC), whereas AIM2 knockout resulted in the opposite effects. This suggested that the AIM2 inflammasome pathway played a critical role in modulating the immune response in dental pulp (57). Furthermore, activation of the AIM2 inflammasome in THP-1 macrophages by *porphyromonas gingivalis* infection had been implicated in the pathogenesis of periodontitis (58). In a model of nonalcoholic fatty liver disease (NAFLD) induced by a high-fat diet (HFD) in mice, mitochondrial DNA (mtDNA) damage was found to activate the AIM2 inflammasome, leading to hepatocyte pyroptosis (59). Notably, the activation of inflammasomes, including both AIM2 and NLRP3, was also implicated in Caspase-1-dependent mitochondrial damage (59). The administration of the AIM2 inflammasome inhibitors resulted in a reduction of inflammation and pyroptosis in the livers of NAFLD-afflicted mice (59). In a similar manner, chronic kidney disease (CKD), which was characterized by inflammation and renal fibrosis, involved AIM2-mediated inflammasome activation and endoplasmic reticulum stress (ERS), both of which played crucial roles in renal fibrosis. Experimental knockout of AIM2, both *in vitro* and *in vivo*, had been shown to attenuate these effects, suggesting that silencing AIM2 disrupted the inflammasome-mediated signaling pathway, thereby mitigating ERS alterations and *in vivo* fibrosis (60). These findings may offer novel therapeutic targets for addressing the pathogenesis of CKD.

**Figure 3:**
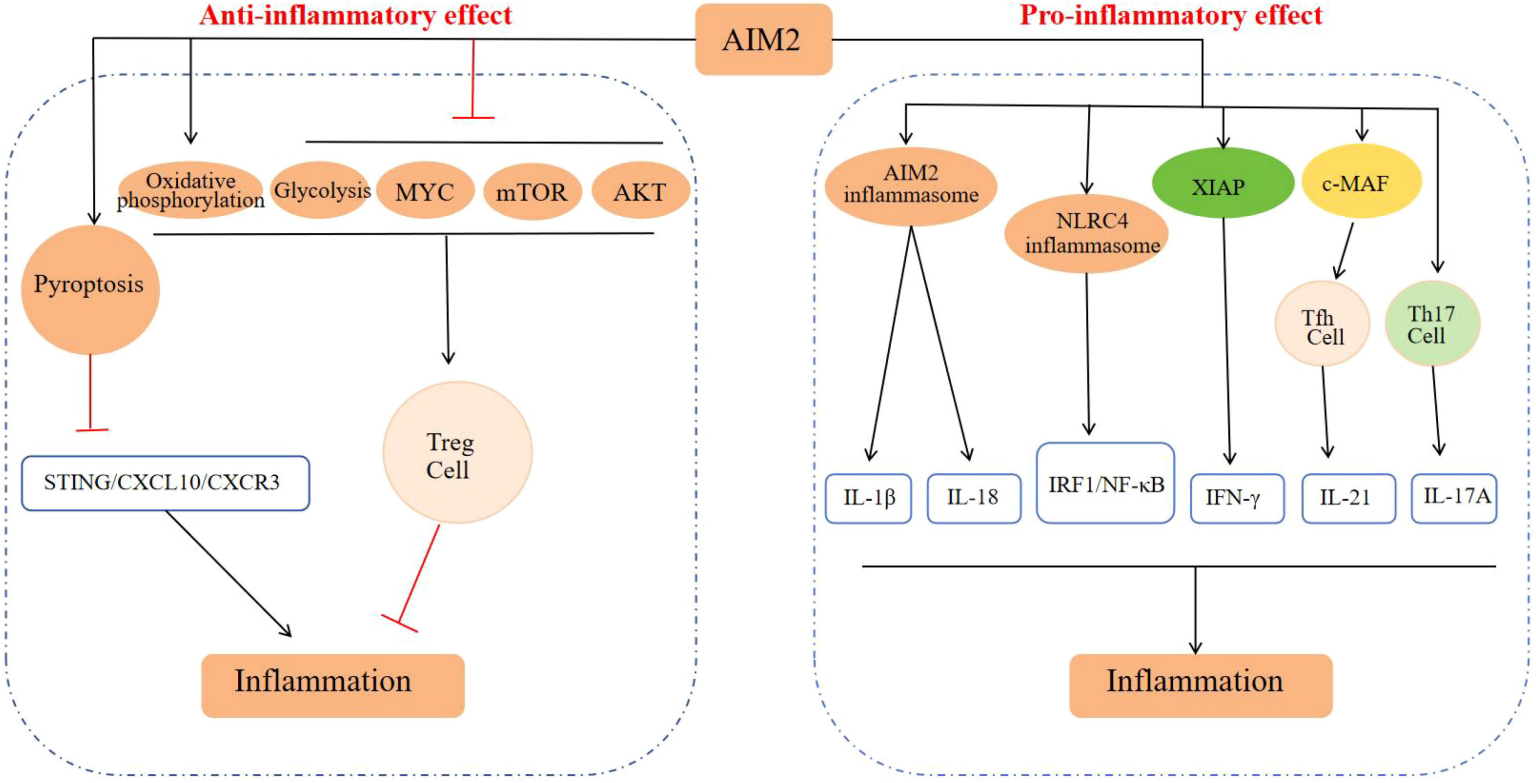
Regulatory mechanism of AIM2 in inflammatory response. In the pro-inflammatory aspects: The AIM2 inflammasome activation leads to the secretion of IL-1β and IL-18, and activation of NLRC4 inflammasome and its downstream IRF1/NF-κB signaling pathway, playing a pro-inflammatory role. AIM2 mediates inflammatory response by enhancing the expression of XIAP and promoting the secretion of IFN-γ. AIM2 can also promote the differentiation of Tfh cells and Th17 cells to produce IL-21 and IL-17A to enhance inflammatory response. In the anti-inflammatory aspects: AIM2 suppresses inflammation by enhancing Treg cell stability and inducing pyroptosis.

In summary, AIM2 is capable of mediating both pro-inflammatory and anti-inflammatory responses via distinct signaling pathways during inflammatory processes. These effects are potentially influenced by the specific cell types in which AIM2 is expressed and the surrounding microenvironment (**Figure 3**).

## 4 Role of AIM2 in tumor

### 4.1 The anti-tumor role of AIM2 in tumors

AIM2 is minimally expressed in melanoma and was originally identified as a tumor suppressor gene, exerting anti-tumor effects through both inflammasome-dependent and independent signaling pathways in tumors. Recent studies had demonstrated that AIM2 expression was significantly reduced in patients with high-grade bladder cancer (BLCA) and was correlated with improved survival rates in these individuals. Moreover, in mice inoculated with cells overexpressing AIM2, there was a notable delay in tumor growth, an extension in survival rates, increased infiltration of CD11b^+^ cells compared to control groups, and heightened activation of the inflammasome within the AIM2 overexpressing cells. BCG immunotherapy demonstrated greater efficacy in tumors exhibiting elevated AIM2 expression (61). These observations elucidate the role of AIM2 inflammasome activation in mitigating cancer progression in BLCA cells and propose that AIM2 inflammasome activation could be leveraged in developing immunotherapeutic strategies for BLCA. A separate investigation involving various cancer cell lines revealed that AIM2 was expressed at low levels in osteosarcoma cell lines. Notably, the overexpression of AIM2 resulted in the inhibition of proliferation, invasion, migration, and epithelial-mesenchymal transition (EMT) in osteosarcoma cells, while simultaneously promoting pyroptosis. Additionally, a significant downregulation of phosphorylated PI3K, AKT, and mTOR levels was observed following AIM2 overexpression. The application of activators targeting the PI3K/AKT/mTOR signaling pathway effectively reversed the impact of AIM2 overexpression on the proliferation, apoptosis, invasion, migration, and EMT of osteosarcoma cells (62). The findings suggest that AIM2 suppresses osteosarcoma progression through the inactivation of the PI3K/AKT/mTOR signaling pathway.

In a similar vein, the expression of AIM2 in human hepatocellular carcinoma (HCC) tissues was markedly lower than in adjacent normal tissues, and patients with HCC exhibiting higher AIM2 expression demonstrated significantly improved overall survival rates. Furthermore, ectopic expression of AIM2 in HCC cells significantly inhibited cellular migration and enhanced apoptosis (63). Additionally, the expression of genes related to the Notch signaling pathway was diminished in the experimental group with AIM2 overexpression, suggesting that Notch signaling might play a role in the regulation of AIM2 within the HCC cellular network (63). Regrettably, this study did not clarify the precise regulatory mechanism of AIM2 within the Notch signaling pathway. Nonetheless, AIM2 had been shown to limit the development of colitis-associated tumors by inhibiting DNA-PK-mediated AKT activation (64), to regulate tumor progression by suppressing intestinal cell proliferation (65), and to mitigate or lower the risk of colorectal cancer through modulation of the gut microbiota (65).

### 4.2 The pro-tumor role of AIM2 in tumors

Nevertheless, AIM2 may also function as a pro-tumorigenic gene, facilitating tumor progression through both inflammasome-dependent and -independent signaling pathways. In oral squamous cell carcinoma (OSCC), the activation of the AIM2 inflammasome initiated pyroptosis and autophagy, concurrently inhibiting p53 protein levels, thereby reducing apoptosis and facilitating cancer cell proliferation (66). Furthermore, AIM2 activation also induced the IL-1β/STAT1/NF-κB signaling pathway via the inflammasome mechanism, which enhanced the expression of programmed death ligand 1 (PD-L1) and contributed to increased radiation resistance and migratory capacity of OSCC cells (67). Recent research had demonstrated that elevated expression levels of AIM2 in patients with lung adenocarcinoma (LUAD) were indicative of a poor prognosis. Subsequent investigations had revealed that the upregulation or activation of the AIM2 inflammasome might facilitate the activation of NF-κB and STAT1 by increasing the levels of IL-1β and IL-18. This process enhanced EMT progression and the expression of PD-L1, ultimately contributing to metastatic progression and immune evasion in LUAD (68). Unfortunately, the specific mechanisms responsible for the upregulation of PD-L1 expression, as well as the existence of alternative bypass regulatory pathways, remain inadequately understood. Subsequent research identified that AIM2 enhanced PD-L1 expression by modulating M2 macrophage polarization and the JAK/STAT3 signaling pathway, thereby inhibiting the immune infiltration of CD8^+^ T cells and preventing CD8^+^ T cell-induced apoptosis of tumor cells, ultimately facilitating immune evasion in LUAD (69). Notably, this study did not elucidate whether the AIM2 inflammasome contributes to these pro-tumor effects.

**Figure 4:**
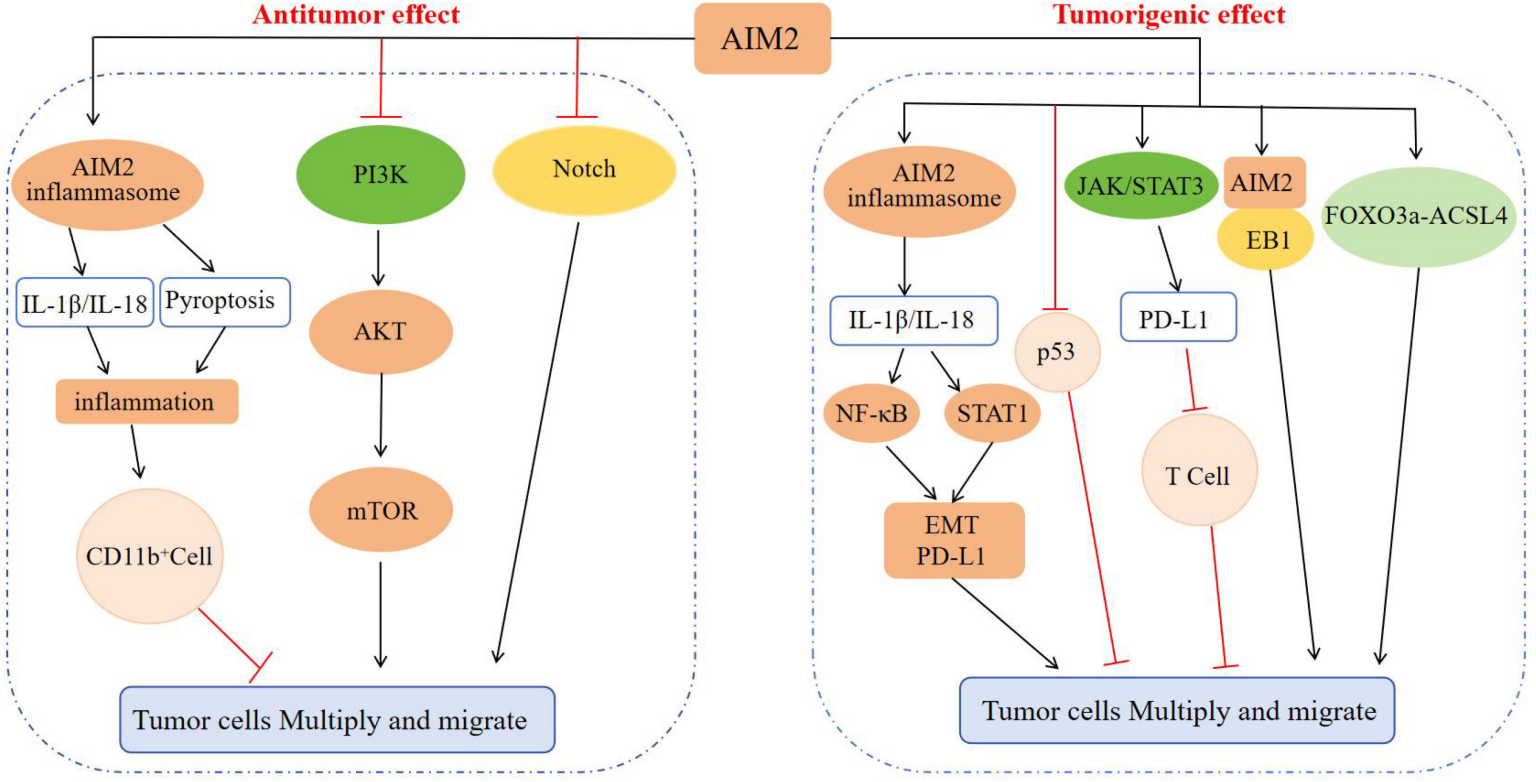
Regulatory mechanism of AIM2 in tumor cell proliferation and migration. In terms of inhibiting tumors: AIM2 causes inflammation through the inflammasome signaling pathway, recruits CD11b^+^ cell infiltration, and inhibits tumor progression; AIM2 can also inhibit tumor cell proliferation, invasion, and migration by inactivating the PI3K/AKT/mTOR signaling pathway and Notch signaling pathway, inhibiting DNA-PK mediated AKT activation, inhibiting intestinal cell proliferation, and regulating intestinal microbiota. In terms of promoting tumors: AIM2 activates NF-κB and STAT1 signaling through inflammasome signaling, promotes EMT progression and enhances PD-L1 expression, or inhibits the anti-tumor effect of p53 protein, or enhances PD-L1 expression through JAK/STAT3 signaling, and then suppresses T cells to promote tumor cell escape, or interacts with EB1 or through FOXO3a-ACSL4 axis regulation, ultimately promoting tumor proliferation and migration.

Previous research had demonstrated that the pro-tumorigenic effects of AIM2 are not necessarily contingent upon inflammasome activation. For instance, in gastric cancer (GC), overexpression of AIM2 had been associated with an increased tumor burden in xenografts derived from human GC cell lines. Subsequent *in vivo* and *in vitro* studies revealed that AIM2 interacted with microtubule-associated end-binding protein 1 (EB1) independently of inflammasome activity, thereby facilitating epithelial cell migration and tumorigenesis (70). Notably, in GC mouse models and the tumor epithelium of GC patients, the IL-11-mediated activation of the oncogenic latent transcription factor STAT3 was found to upregulate AIM2 expression (70). These studies demonstrated that AIM2 facilitates the progression of epithelial carcinogenesis by linking cytokine STAT3 signaling with epithelial cell migration, independently of the inflammasome signaling pathway. Parallel investigations had also revealed that AIM2 advanced renal cell carcinoma (RCC) progression through the modulation of the FOXO3a-ACSL4 axis in the context of iron deficiency anemia, again independent of inflammasome pathways (71). Collectively, these findings offer novel insights into the role of AIM2 in oncological research (**Figure 4**).

## 5 Conclusion

Under typical conditions, the intracellular receptor AIM2 remains in a quiescent state. Upon detection of exogenous dsDNA or its analogues, AIM2 becomes activated and can exert either pro-inflammatory or anti-inflammatory effects in the context of inflammation and tumorigenesis. This is mediated through the inflammasome signaling pathway or other associated AIM2-related signaling pathways. Notably, the specific role of AIM2 is contingent upon the cell type in which it is expressed and the nature of the host disease, whether involving exogenous or mutated dsDNA (**Table 1**).

**Table 1:** The role of AIM2 in inflammation and tumors.

Category	Diseases	Mode	Mechanism	Function	Cells	Infla-mmas-ome	Ref.
**Autoimmune systemic inflammation**	RA	Clinical research	AIM2 might damage articular cartilage by producing inflammatory cytokines.	Promotion	FLS	Yes	(17)
		Clinical and *in vivo* research	AIM2 induced AKT phosphorylation leading to RA inflammation.	Promotion	FLS	Not clarified	(26)
	Gouty arthritis	*In vivo* and *in vitro* research	The AIM2 inflammasome mediated inflammation and pyroptosis promoted pain and inflammation in gouty arthritis.	Promotion	PBMCs THP-1 Macropha-ges(Mφs)	Yes	(22)
	pSS	*In vivo* and *in vitro* research	The AIM2 inflammasome and STING induced proinflammatory responses in lacrimal gland myoepithelial cells (MECs).	Promotion	MECs	Yes	(23)
	Psoriasis	*In vivo* and *in vitro* research	NETs-AIM2 axis exerted multiple pro-inflammatory effects on keratinocytes.	Promotion	Keratinocytes	Yes	(27)
	SLE	*In vivo* and *in vitro* research	AIM2 promoted Tfh cell differentiation and IL-21 production through the c-MAF signaling pathway, promoting the development process of SLE.	Promotion	Tfh cells	No	(28)
	Rhabdomyolysis	*In vivo* and *in vitro* research	AIM2 induced pyroptosis to limit renal inflammation due to rhabdomyolysis.	Inhibition	Mφs in the kidney	No	(33)
**Head disease inflammation**	DED	*In vivo* and *in vitro* research	Aim2 increased scopolamine (SCOP) induced ocular surface damage and Lacrimal gland (LG) inflammation in DED.	Promotion	Mφs	Yes	(24)
	EAU	*In vivo* and *in vitro* research	The AIM2 inflammasome mediated EAU.	Promotion	Mφs	Yes	(25)
	Pulpitis	Clinical and *in vitro* research	The AIM2 inflammasome activation promoted the development of pulpitis.	Promotion	Human dental pulp cells (HDPCs)	Yes	(58)
	Periodontitis	*In vivo* and *in vitro* research	The AIM2 inflammasome activation promoted the development of periodontitis.	Promotion	THP-1 Mφs	Yes	(59)
**Cardiovascular and cerebrovascular system inflammation**	Atherosclerosis	*In vivo* and *in vitro* research	AIM2 promoted the development of atherosclerosis by enhancing endothelial cells migration and apoptosis.	Promotion	Human coronary artery endothelial cells (HCAEC)	Yes	(38)
			AIM2 regulated VSMCs migration, leading to atherosclerosis.	Promotion	VSMCs	Yes	(39)
			The AIM2 inflammasome exacerbated atherosclerosis.	Promotion	Mφs	Yes	(40)
	CAD	Clinical and *in vitro* research	The AIM2 inflammasome activation directed monocytes to an inflammatory state or even pyroptosis, which was associated with the development of CAD.	Promotion	Monocytes	Yes	(42)
	Diabetic cardiomyopathy	*In vivo* and *in vitro* research	Activation of the AIM2 inflammasome led to cardiac hypertrophy and remodeling.	Promotion	Mφs	Yes	(44)
	Stroke	*In vivo* and *in vitro* research	AIM2 deficiency prevented ischemia-induced BBB damage by downregulating ICAM-1 expression.	Promotion	Brain endothelial cells(BECs)	Not clarified	(45)
			Activation of the AIM2 inflammasome aggravated ischemic stroke.	Promotion	Astroglia and microglia	Yes	(46)
			Activation of the AIM2 inflammasome caused neuronal pyroptosis.	Promotion	Neurons	Yes	(48)
			The AIM2 inflammasome activation led to excessive T cell death to aggravate stroke disease.	Promotion	Bone Marrow-Derived Macrophages (BMDM) and T cell	Yes	(50)
			Activation of the AIM2 inflammasome aggravated IS.	Promotion	Microglia/Mφs	Yes	(51)
			Activation of the AIM2 inflammasome aggravated BBB disruption.	Promotion	Neutrophils	Yes	(52)
**Urinary system inflammation**	CKD	*In vivo* and *in vitro* research	The AIM2 inflammasome mediated inflammation activation and ERS in the process of aldosterone-induced renal injury.	Promotion	Glomerular mesangial cells and Tubular epithelial cells	Yes	(60)
**Digestive system inflammation**	Hepatic steatosis	*In vivo* and *in vitro* research	AIM2 inflammasome released IL-18 and IL-1 β after activation, which promoted hepatic steatosis.	Promotion	Liver cells	Yes	(59)
	Colitis	*In vivo* and *in vitro* research	AIM2 promoted Th17 cell differentiation and mediated the development of colitis by regulating RORγt transcriptional activity.	Promotion	Th17 cells	No	(15)
			AIM2 enhanced Treg cell stability by reducing AKT-mTOR signaling and modifying immune metabolism, thereby alleviating autoimmune diseases.	Inhibition	Treg cells	No	(32)
**Nervous system inflammation**	AD	*In vivo* and *in vitro* research	Activation of the AIM2 inflammasome increased neuroinflammation.	Promotion	Microglia	Yes	(53)
	EAE	*In vivo* and *in vitro* research	AIM2 negatively regulated DNA-PK-AKT3 in microglia to control neuroinflammation induced by cGAS and DNA-PK.	Inhibition	Microglia	No	(54)
	PD	*In vivo* and *in vitro* research	AIM2 reduced cGAS mediated antiviral inflammation by inhibiting IRF3 phosphorylation.	Inhibition	Microglia	No	(55)
**Urinary system** **tumors**	BLCA	*In vivo* and *in vitro* research	The AIM2 inflammasome activation might inhibit the BLCA tumorigenesis and enhance the therapeutic effect of BCG in BLCA.	Inhibition	BLCA cells	Yes	(61)
	RCC	Clinical and *in vitro* research	AIM2 promoted RCC progression through FOXO3a-ACSL4 axis-regulated ferroptosis.	Promotion	RCC cells	No	(71)
**Digestive system tumors**	HCC	Clinical and *in vitro* research	AIM2 inhibited the growth and metastasis of HCC.	Inhibition	HCC cells	No	(63)
	Colorectal cancer	*In vivo* and *in vitro* research	AIM2, in an inflammasome-independent manner, inhibited DNA-PK mediated Akt activation and reduced colorectal tumorigenesis.	Inhibition	Mφs	No	(64)
			AIM2 controlled the proliferation of intestinal epithelial cells through mechanisms independent of inflammasome and inflammatory mediators, thereby protecting them from tumorigenesis.	Inhibition	Intestinal epithelial cells	No	(65)
	GC	*In vivo* and *in vitro* research	AIM2 might play a driver role in epithelial carcinogenesis by linking cytokine-STAT3 signalling, innate immunity and epithelial cell migration, independent of inflammasome activation.	Promotion	GC cells	No	(70)
	OSCC	*In vitro* research	The AIM2 inflammasome activation initiated the cell pyroptosis pathway, suppressed p53 protein, suppressed cell apoptosis, and promoted cancer cell proliferation.	Promotion	OSCC cells	Yes	(66)
			The AIM2 inflammasome activation triggered the IL-1β/STAT1/NF-κB pathway, enhanced the expression of PD-L1 and promoted the radio-resistance and migration ability of OSCC.	Promotion	OSCC cells	Yes	(67)
**Respiratory system tumors**	LUAD	*In vitro* research	AIM2 promoted the activation of NF-κB and STAT1 through IL-1β and IL-18 produced by the activated inflammasome, thus enhancing the expression of PD-L1, which ultimately led to the metastatic progression and immune evasion of LUAD.	Promotion	LUAD cells	Yes	(68)
			AIM2 promoted LUAD by regulating M2 macrophage polarization and upregulating PD-L1 expression through the JAK/STAT3 signaling pathway.	Promotion	Mφs	Not clarified	(69)
**Skeletal system tumors**	Osteosarcoma	*In vitro* research	AIM2 inhibited the progression of osteosarcoma by inhibiting PI3K/AKT/mTOR signaling pathway.	Inhibition	Osteosarc-oma cells	No	(62)

The influence of AIM2 offers new insights into the mechanisms underlying inflammation and tumors progression, thereby laying the groundwork for the development of advanced clinical immunotherapies. Targeting AIM2 as a therapeutic intervention may yield varying outcomes across different clinical diseases. By considering the specific advantages and disadvantages of AIM2 in disease contexts, timely and targeted AIM2 therapies could result in favorable therapeutic outcomes. Furthermore, elucidating the complementary interactions between AIM2 and other DNA receptors across various cell types, tissues, and organ contexts can enhance our comprehensive understanding of the biological characteristics of AIM2. This knowledge may contribute to improved host immune surveillance in the context of autoimmune diseases, infectious diseases, and cancer, while simultaneously mitigating adverse inflammatory responses and autoimmune conditions that may compromise human health.
